# Denoising of 3D Brain MR Images with Parallel Residual Learning of Convolutional Neural Network Using Global and Local Feature Extraction

**DOI:** 10.1155/2021/5577956

**Published:** 2021-05-04

**Authors:** Liang Wu, Shunbo Hu, Changchun Liu

**Affiliations:** ^1^School of Control Science and Engineering, Shandong University, Jinan 250061, China; ^2^School of Information Science and Engineering, Linyi University, Linyi 276005, China

## Abstract

Magnetic resonance (MR) images often suffer from random noise pollution during image acquisition and transmission, which impairs disease diagnosis by doctors or automated systems. In recent years, many noise removal algorithms with impressive performances have been proposed. In this work, inspired by the idea of deep learning, we propose a denoising method named 3D-Parallel-RicianNet, which will combine global and local information to remove noise in MR images. Specifically, we introduce a powerful dilated convolution residual (DCR) module to expand the receptive field of the network and to avoid the loss of global features. Then, to extract more local information and reduce the computational complexity, we design the depthwise separable convolution residual (DSCR) module to learn the channel and position information in the image, which not only reduces parameters dramatically but also improves the local denoising performance. In addition, a parallel network is constructed by fusing the features extracted from each DCR module and DSCR module to improve the efficiency and reduce the complexity for training a denoising model. Finally, a reconstruction (REC) module aims to construct the clean image through the obtained noise deviation and the given noisy image. Due to the lack of ground-truth images in the real MR dataset, the performance of the proposed model was tested qualitatively and quantitatively on one simulated T1-weighted MR image dataset and then expanded to four real datasets. The experimental results show that the proposed 3D-Parallel-RicianNet network achieves performance superior to that of several state-of-the-art methods in terms of the peak signal-to-noise ratio, structural similarity index, and entropy metric. In particular, our method demonstrates powerful abilities in both noise suppression and structure preservation.

## 1. Introduction

Medical image information is playing an increasingly important role in disease diagnosis. However, during the image acquisition process, due to the improper actions of patients or staff, strong random noise will inevitably be generated. This noise not only reduces the resolution of the image but also affects the precision of clinician diagnosis [1, 2].

At present, popular magnetic resonance (MR) imaging technology is commonly used as a medical imaging technology for visualizing human tissues and organs. It does not pose any radiation hazard, unlike CT imaging [3], and it achieves multiaspect, multiparameter, and high-contrast-resolution images without bone artifacts. However, the random noise will affect the inspection quality in clinical diagnosis, as well as image processing and analysis tasks such as image segmentation, registration, and visualization. Hence, solving the problem of MR image denoising is critical.

The purpose of image denoising is to remove background noise and retain valuable information [4]. Many conventional filtering techniques are often used, such as Wiener filtering [5], bilateral filtering [6], and total variation filtering [7]. Yang and Fei proposed a multiscale wavelet denoising method based on the Radon transform to denoise MR images [8]. Phophalia et al. mitigated the problem of medical image denoising by using rough set theory (RST) [9]. Awate and Whitaker devised a Bayesian denoising method and verified it on diffusion-weighted MR images [10]. Satheesh et al. developed an MR image denoising algorithm using the contourlet transform, which achieved a higher peak signal-to-noise ratio than the wavelet transform [11]. Zhang et al. used an improved singular value decomposition method to denoise simulated and real 3D images. The experimental results showed that their method was superior to the existing denoising methods [12]. Leal et al. presented a method based on sparse representations and singular value decomposition (SVD) for nonlocally denoising MR images. This method prevents blurring, artifacts, and residual noise [13]. In addition, by extending the local region to a nonlocal scheme, the nonlocal means (NLM) strategy was used for MR image denoising [14–16]. Gautam et al. proposed a novel denoising technique for MR images based on the advanced NLM method with non-subsampled shearlet transform (NSST) [17]. Kanoun et al. proposed an enhanced NLM filter using the Kolmogorov-Smirnov (KS) distance. The experimental results provided excellent noise reduction and image-detail preservation [18].

In recent years, the explosive development of deep learning has suggested a new methodology for image denoising. It can use multiple convolution filters to automatically extract features, with large receptive fields, to reconstruct high-resolution images. In [19], the authors used the self-encoder to train the image features of different resolutions to achieve adaptive denoising. Zhang et al. exploited denoising convolutional neural networks (DnCNNs) for Gaussian noise removal and achieved excellent performance by using residual learning strategy [20]. Cherukuri et al. applied a deep learning network that leveraged the prior spatial structure of images to reconstruct high-resolution images [21]. Manjo′n et al. proposed a novel automatic MR image denoising method by combining a convolutional neural network (CNN) with a traditional filter [22].

Deep learning-based denoising methods can grasp richer contextual information in large regions to improve performance. With very deep architectures, it can expand the receptive field of the network to capture more global contextual information over large image regions. Liu et al. utilized the multiscale fusion convolution network (MFCN) to perform super-resolution reconstruction of MR images [23]. Pham et al. used a deep 3D CNN model with residual learning to reconstruct MR images [24]. Their model exploited a very deep architecture with a large receptive field to acquire a powerful learning ability. Jiang et al. described a multichannel denoising convolutional neural network (MCDnCNN) that directly learned the process of denoising and performed experiments on simulation and real MR data [25]. In [26], Ran et al. suggested a residual encoder-decoder Wasserstein generated countermeasure network (RED-WGAN) for MR image denoising. Hong et al. designed a spatial attention mechanism to obtain the area of interest in MR images, which made use of the multilevel structure and boosted the expressive ability of the network [27]. Tripathi and Bag proposed a novel CNN for MR image denoising. The proposed model consisted of multiple convolutions that captured different image features while separating inherent noise [28]. Li et al. designed a progressive network learning strategy by fitting the distribution of pixel-level and feature-level intensities. Their experimental results demonstrated the great potential of the proposed network [29]. Gregory et al. created HydraNet, a multibranch deep neural network architecture that learned to denoise MR images at a multitude of noise levels, and proved the superiority of the network on denoising complex noise distributions compared to some deep learning-based methods [30]. Aetesam and Maji proposed a neural framework for MR images denoising, using an ensemble-based residual learning strategy. High metric value and high-quality visual results were obtained in both synthetic and real noisy datasets [31].

In the above reported deep learning denoising tasks, the depth and width of the networks were often increased to capture more contextual information. However, these methods introduced a number of parameters, which made it difficult to train the denoising models. Some of the methods learned the Rician noise distribution solely by stacking convolution layers, which easily overlooked much local information and led to unsatisfactory denoising results at some key local anatomical positions.

To address the above shortcomings, this work proposes a novel network termed 3D-Parallel-RicianNet that is used to remove the noise of MR images. First, to expand the receptive field without introducing more parameters, we design a dilated convolution residual (DCR) module and use it to build a subnetwork (DCRNet) that can extract global information by cascading. Then a depthwise separable convolution residual (DSCR) module is designed and used to construct a subnetwork (DSCRNet) to extract local information. Finally, the features of each module of DCRNet and DSCRNet are merged and cascaded to obtain full-scale mappings between image appearances and noise deviation.

The main contributions of this work are summarized as follows:

1. DCRNet expands the receptive field to extract rich context information through cascading DCR modules, which capture the real Rician distribution in the global area
2. DSCRNet uses the DSCR module to focus on the local area of the image and effectively removes local anatomical noise. Each DSCR module of this subnetwork is added to the output part of each DCR module of the corresponding DCRNet
3. The 3D-Parallel-RicianNet uses a residual learning mechanism to prevent vanishing and exploding gradient problems

The remainder of this work is organized as follows. In Section 2, we describe the proposed denoising networks and loss function. Then, in Section 3, we present the experimental tests of our approach on synthetic and real MR noisy data. Additionally, a comparison of our method with state-of-the-art algorithms is provided. Finally, in Section 4, we discuss our conclusions and give future directions.

## 2. Materials and Methods

### 2.1. Noise Reduction Model

**Figure 1:**
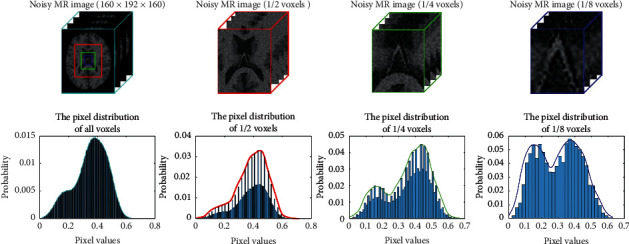
PDFs in different sizes of subwindows. The height of each vertical bar is the proportion of the corresponding pixel value. The lines represent the fitted pixel distribution. Top row from left to right: a 3D T1-weighted MR image with 7% Rician noise, a 1/2-voxel T1-weighted MR image, a 1/4-voxel T1-weighted MR image, and a 1/8-voxel T1-weighted MR image. Bottom row PDFs are of intensity in the corresponding voxels. As shown, the PDF (1/2 voxels) within the red region tends to be similar for the whole image. However, the PDFs in the small local green region (1/4 voxels) and local blue region (1/8 voxels) are different from those in the global region.

MR magnitude image is corrupted by independent Gaussian distribution noise in the real part and the imaginary part of images [32–34]. Previous studies suggest that the probability distribution of noisy MR image pixel intensity can be represented as a Rician distribution [35, 36]. Deep learning can ignore the physical process and model this procedure corruption by learning from the samples [26]. Hence, the MR image degradation model with noise can be described as

(1) $$\begin{array}{c} Y=X+\delta \left(Y\right), \end{array}$$

where *Y* is the noisy MR image, *X* is the noise-free image, and *δ*(*Y*) is the deviation between *X* and *Y* influenced by the Rician distribution. According to equation (1), *δ*(*Y*) can be expressed as (*Y* − *X*), so it was employed to train a residual mapping *f*(*Y*; Θ) ≈ *δ*(*Y*), and we can obtain *X* ≈ *Y* − *f*(*Y*; Θ).  Figure 1 shows that the probability density distribution (PDF) of noisy MR images varies in global and local regions. It can be seen from the top left image that the noise reduces the quality of the MR image and blurs the boundaries of some tissue structures, which results in increased difficulty in recognizing the image details. Liu et al. pointed out that the PDFs of Rician noise vary spatially in different anatomical regions of brain MR images [37]. Hence, the nonlinear mappings between image appearances and Rician distributions vary in global and local regions. Based on this conclusion, we propose the 3D-Parallel-RicianNet MR image denoising model, which combines the global and local feature information on global regions and local regions.

### 2.2. DCR Module for Global Feature Representation

It is known that context information is important to reconstruct corrupted pixels for image denoising. Specifically, it is a common way to capture more global context information by expanding the receptive field [38]. In the reported deep learning denoising tasks, increasing the depth and width of the deep networks can enlarge the receptive field. However, the width-adding methods may produce more parameters, which results in overfitting of the network. The depth-adding methods may lead to vanishing gradients when the depth of the network is enormous.

**Figure 2:**
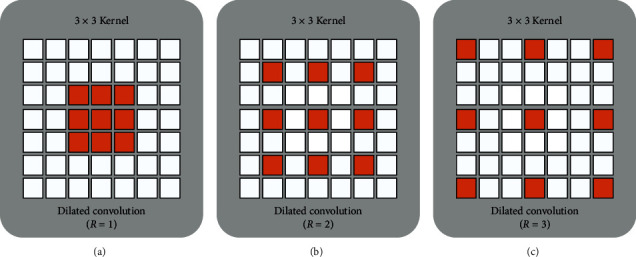
Dilated convolution. (a) The receptive field of the convolution kernel of size 3 × 3 pixels, which covers a 3 × 3 subregion in the image through a convolution operation. (b) The receptive field of the convolution kernel with an *R* of 2. (c) The receptive field of the convolution kernel with an *R* of 3.

To solve these problems, dilated convolutions have been developed [39]. The dilation rate of the convolution kernel can be controlled to obtain receptive fields of different sizes, as shown in Figure 2. The size of the receptive field, *v*, is denoted as

(2) $$\begin{array}{c} v={\left(\left(k_{\text{size}}-1\right)\times \left(R-1\right)+k_{\text{size}}\right)}^{d}, \end{array}$$

where *k*_size_ is the size of the filter, *R* is the dilated rate, and *d* is the dimension (2 or 3) of the image. The receptive field of the convolution operation can be expanded by setting different *R*. This creates a tradeoff between increasing the depth and width of CNNs. In [40], Peng proposed dilated residual networks with symmetric skip connection (DSNet). The experiments demonstrated that the model was more feasible for the task of image denoising, especially for Gaussian noise. Zhang et al. proposed a dual-domain multiscale CNN (DMCNN) for JPEG artifacts based on dilated convolution. This also proved that dilated convolution had advantages in restoring image quality [41].

**Figure 3:**
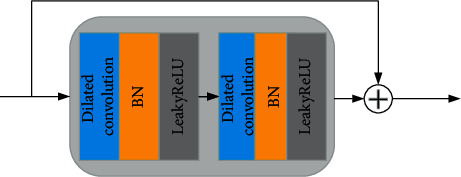
The structure of the DCR module.

In this study, we construct the DCR module as one component of our 3D-Parallel-RicianNet. It exploits dilated convolutions to extract global features, as shown in Figure 3. The DCR module consists of dilated convolution, residual learning, batch normalization (BN), and leaky rectified linear unit (LeakReLU). Residual learning fundamentally breaks the symmetry of the network, thereby improving the ability of the representation network. By setting the BN layer, the generalization ability of the network is improved. Due to the problem of vanishing gradients using the ReLU activation function, we use LeakReLU as the activation function of the network. The input and output of a two-level dilated convolution are briefly connected to construct a DCR module.

### 2.3. DSCR Module for Local Feature Representation

It is very important to recover the local fine details in image denoising. When some local features are not well extracted, the local denoising effect will be degraded. Recently, depthwise separable convolution (DSConv) has been used in many advanced neural networks, such as Xception [42], MobileNets [43], and MobileNets2 [44], to replace the standard convolutional layer, aiming to reduce CNN computational cost and to extract local features [45].

**Figure 4:**
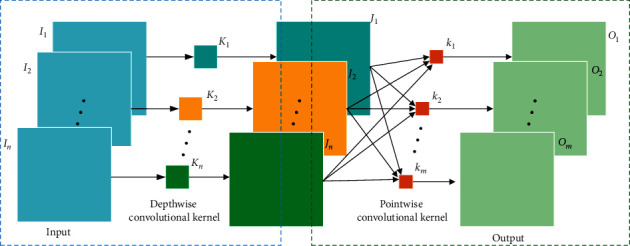
Depthwise separable convolutions (DSConv).

DSConv consists of two parts: depthwise convolution and pointwise convolution. As shown in Figure 4, the depthwise convolution acts on each input channel separately, to exact local features, followed by a pointwise convolution that uses 1 × 1/1 × 1 × 1 convolution to weight the features among channels at every point. Hence, this would efficiently extract the local features among different channels. The input feature map is *I*={*I*_1_, *I*_2_,…, *I*_*n*_}. First, using depthwise convolutions with *n* filters *K*={*K*_1_, *K*_2_,…, *K*_*n*_}, an intermediate result *J*={*J*_1_, *J*_2_,…, *J*_*n*_} is produced, which is then processed into the output feature map *O*={*O*_1_, *O*_2_,…, *O*_*m*_} by means of the pointwise convolutions using *m* filters *k*={*k*_1_, *k*_2_,…, *k*_*m*_}.

DSConv can extract local delicate features of the image by considering the information of the position and channel separately. Imamura et al. designed a denoising network for hyperspectral images using DSConv and demonstrated its ability to realize efficient restoration [46]. The advantage of DSConv is that it reduces the number of network parameters and the computational complexity in convolution operations [42–44].

**Figure 5:**
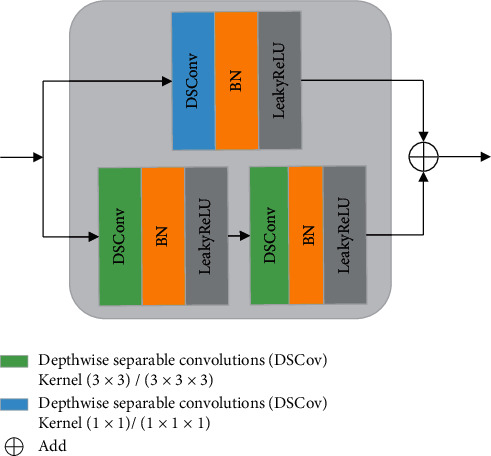
The structure of the DSCR module.

The model designed by using dilated convolution can restore the image quality globally [40, 41] but can easily ignore local information. To solve this problem, inspired by DSConv, we extend the technique to the DSCR module to extract the local information of the MR images, as shown in Figure 5. We utilize the residual strategic idea and take the depthwise separable convolutions as the main construction module. On the one hand, we design two continuous depthwise separable convolutions with the BN layer after each convolution layer to improve the generalization ability of the network. On the other hand, we use another depthwise separable convolution to shortcut the module to prevent vanishing gradients.

### 2.4. The Proposed 3D-Parallel-RicianNet Model

**Figure 6:**
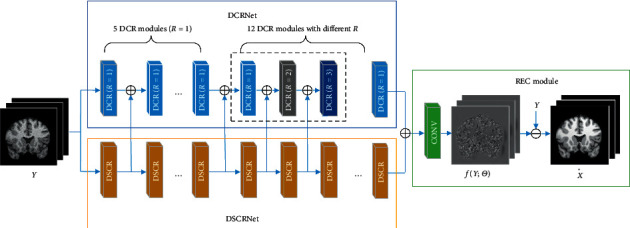
The proposed network architecture for MR image denoising.

The proposed 3D-Parallel-RicianNet framework consists of a global feature extraction network DCRNet, a local feature extraction network DSCRNet, and a reconstruction (REC) module. Under this framework, the pipeline of MR image denoising is composed of three major steps (see Figure 6). First, we apply DCRNet and DSCRNet to extract the global features and local features, respectively. Then, we fuse the global and local features through an additional layer to obtain real Rician distribution features. Finally, we use the REC module to obtain a predicted clean MR image $\hat{X}$.

*DCRNet*. The proposed DCRNet framework is a cascade of 18 DCR modules with different *R*. The kernel size is 3 × 3/3 × 3 × 3 for 2D slices/3D patches. Dilated convolution with a large *R* behaves well for low-frequency noise removal. When *R* is too large, it is difficult to capture some small contextual information, which will cause the waste of receptive fields. If *R* is 1, it is the same as the traditional convolution in each channel. In DCRNet, to ensure that all feature maps have the same size as the input, we symmetrically pad zeros around the boundaries before applying the convolution operation. As the convolutional layer increases, the range of the receptive field will gradually increase. In addition, a gridding problem is known to exist in dilated convolution [47]. To solve these problems, considering the size of the input in our experiments, we applied DCR modules with different dilation rates. Therefore, the dilated rate of each layer is set to 1, 1, 1, 1, 1, 1, 2, 3, 1, 2, 3, 1, 2, 3, 1, 2, 3, and 1. The final receptive field is 61. Multiscale global features are extracted by using multiple DCR modules with different dilation rates. Each module has 16 filters. The implementations can avoid the gridding effects and reduce the influence of unrelated information.

*DSCRNet*. DSCRNet is further used to compensate for the local information ignored by expanding the receptive field. It is a cascade of 18 DSCR modules. The size of the convolution kernel of each module is 3 × 3/3 × 3 × 3. Each module also has 16 filters.

We fused the features extracted from each module of DCRNet and DSCRNet to gradually realize the complementarity of global and local information. This process particularly helps to preserve critical image features in global regions and local regions. Therefore, the proposed 3D-Parallel-RicianNet model will have better denoising ability than other methods.

*REC* Module. After a convolution layer, we obtain the estimated deviation *f*(*Y*; Θ) and then use $\hat{X}=Y-f\left(Y;\Theta \right)$ to obtain a predicted clean MR image.

### 2.5. Loss Function

Our loss function uses the mean squared error (MSE) as follows:

(3) $$\begin{array}{c} \iota \left(\Theta \right)=\frac{1}{N}\sum_{i=1}^{N}Y_{i}-f\left(Y_{i};\Theta \right)-X_{i}^{2}. \end{array}$$

where *X*_*i*_ is the *i*^th^ noise-free image, *Y*_*i*_ is the corresponding noisy image, and Θ denotes the network parameters. We minimize this loss function to learn the output noise-free image (*Y*_*i*_ − *f*(*Y*_*i*_; Θ)).

## 3. Experiments Results and Analysis

### 3.1. Dataset Description

To validate the performance of the proposed 3D-Parallel-RicianNet, extensive experiments were performed on both public simulated and clinical datasets.

For simulated experiments, the BrainWeb dataset [48, 49] was used. In this work, we obtained 18 T1-weighted (T1w) MR images with different noise levels (1%, 3%, 5%, 7%, and 9%). The size of the image is 181 × 217 × 181, and its resolution is 1 × 1 × 1mm^3^. The brain skull is stripped by the skull mask. To further speed up the training process and obtain fewer redundant areas, we cropped the edges of the image, and the image size is 160 × 192 × 160.

One critical problem of the deep learning approach is weak generalization applicability. Networks trained on one dataset from a specific manufacturer or setting may not perform well for a different dataset. The noise in the simulation dataset is assumed to come from single coil acquisition systems. However, clinical MR image noise distributions come from multiple coils, and these noises are subject to a noncentral Chi distribution with a sum-of-squares (SoS) reconstruction. Actually, the Rician distribution is a special case of the noncentral Chi distribution [50] and varies spatially in real MR images [37].

To verify the generalization ability of the proposed model, we carried out experiments on real datasets. For the first clinical experiment, the well-known IXI dataset [51] was used, which was collected from 3 different hospitals. We randomly selected 100 T1w brain images from the Hammersmith dataset. The image size is 256 × 256 × 150, and the voxel resolution is 0.9375 × 0.9375 × 1.2mm^3^. Sixty images were randomly selected as the training set, 20 images for validation, and the other 20 images for testing. In this dataset, we manually added different levels of Rician noise to simulate the noisy image [26]. The brain skull was stripped by the VolBrain method [52].

For another experiment, we randomly selected 35 T1w images in ADNI [53]. Each of these samples contained 192 × 192 × 160 voxels with 1.2 × 1.25 × 1.25mm^3^ voxel resolution. For the experiment, the original scan was resized to dimensions of 256 × 256 × 128. The brain skull was also stripped by the VolBrain method. Due to the lack of knowledge about the noise level in real data, we used the variance-stabilization approach to estimate the Rician noise level of ADNI data, which was approximately 3% [54]. Hence, we selected IXI models trained with a 3% noise level to test ADNI data.

The last dataset comes from the Combined Healthy Abdominal Organ Segmentation (CHAOS) challenge [55, 56]. The dataset included 40 abdominal T1w MR images. On average, each volume size is 256 × 256 × 36, and the noise level is unknown. We adjusted the image to 256 × 256 × 64 through zero-padding operations to be uniform. To substantiate the robustness and generalization capability of the proposed framework, we employed this dataset for our experiments, splitting it into subsets of 25, 5, and 10 subjects that were used for training, validation, and testing.

### 3.2. Training Details

We use two strategies for training on the three datasets of BrainWeb, IXI-Hammersmith, and CHAOS: 2D slice-based training and 3D patch-based training. For 2D training, we extracted 2D coronal slices from 3D data in the BrainWeb dataset. We obtained 2880 slices by rotating 90° and mirroring, with 1920 slices for training, 384 slices for validation, and 576 slices for testing. In the IXI-Hammersmith dataset, we cropped the image to 256 × 256 × 128 and tested it in all clinical brain datasets. We extracted 7680 slices for training, 2560 slices for validation, and 2560 slices for testing in the sagittal plane. In the CHAOS dataset, using rotation and mirroring to expand the data, we obtained 6400 sagittal slices for training, 1280 sagittal slices for validation, and 640 sagittal slices for testing.

For patch-based training, 3D data in the BrainWeb dataset was also expanded by rotation and mirroring. To reduce memory burden, we used patches with a size of 64 × 64 × 64 voxels. A sliding window strategy with a stride of 16 × 32 × 16 was then used to obtain 3675 patches to train the 3D model. Using the same strategy as the BrainWeb dataset, 4500 training patches, 1500 validation patches, and 1500 test patches were extracted from IXI-Hammersmith with a step size of 48 × 48 × 32. We used rotating and mirroring to expand the CHAOS dataset before extracting patches and finally obtained 4900 training patches, 980 validation patches, and 490 test patches with a stride of 32 × 32 × 64. In the training stage, since the CHAOS dataset did not have clean images and the noise level was unknown, we used the 5% noise model trained by IXI-Hammersmith to estimate clean images as ground truth. In the testing stage, we applied the trained network to patches of the test set. The resultant predictions were averaged in the overlapping regions.

All training was conducted using a deep learning acceleration computing service, which is configured with a 2.20 GHz Core i7-8750H CPU, an NVIDIA GeForce GTX 1070 (8G) GPU, and 16 GB RAM. All the deep learning models were implemented with the publicly available TensorFlow framework and Keras artificial neural network library. In the training process, the learning rate was set to 1e-3. We used Adam optimization.

### 3.3. Evaluation Methods

Six kinds of deep learning models were trained: CNN-DMRI [28], RicianNet [29], 2D-DCRNet, 2D-Parallel-RicianNet, 3D-DCRNet, and 3D-Parallel-RicianNet. We compared these six deep learning models with four traditional denoising methods: NLM, BM3D, ODCT3D [57], and PRI-NLM3D [57]. In the NLM method, the fastNLMMeansDenoising function is selected, where the template size is 7 × 7 and the filter strength is 15.

Three quantitative metrics were employed to evaluate the denoising performance of these methods. The first was the peak signal-to-noise ratio (PSNR). A high PSNR generally denotes good denoising performance. The second was the structural similarity index (SSIM), which measured the structural similarity between the ground-truth and denoised images. The last one was entropy, which reflected the amount of image information. We used the natural logarithm in the entropy metric.

### 3.4. Simulated Results

The quantitative results of NLM, BM3D, ODCT3D, PRI-NLM3D, CNN-DMRI, 2D-DCRNet, 2D-Parallel-RicianNet, 3D-DCRNet, and 3D-Parallel-RicianNet on T1w images with different noise levels (1%, 3%, 5%, 7%, 9%) are illustrated in Tables 1–3.

Tables 1 and 2 depict the PSNR and SSIM results, respectively. We can observe that the PSNR values of 3D-Parallel-RicianNet are obviously higher than those of the other methods at all noise levels. In Table 2, the SSIM values of 3D-Parallel-RicianNet are closer to 1, which is higher than those of the other methods under all noise levels except PRI-NLM3D at the 7% noise level. This indicates that our proposed model has good denoising performance with good anatomical structure preservation.

Table 3 shows the entropy results of 10 methods. We find that the proposed 3D-Parallel-RicianNet can obtain the lowest entropy under all five noise levels. Hence, considering the three metrics in 3 tables, we find that our method has better noise reduction performance. In addition to visual quality, another important aspect of the MR image denoising method is the time complexity. We give running times for different methods in Table 4. It is clear that 3D-DCRNet and our proposed 3D-Parallel-RicianNet are much faster than other methods. Once the deep learning-based method finishes training, forward propagation is very fast. In Table 5, our method has the fewest parameters, which means that our network does not need too much computational power. From this, we can see that our model has competitive advantages for small data sets.

**Figure 7:**
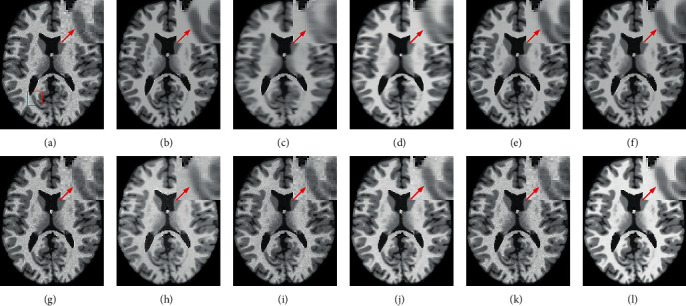
Denoising effect of different methods with 3% noise level. (a) Noisy image; (b) noise-free image; (c) NLM; (d) BM3D; (e) ODCT3D; (f) PRI-NLM3D; (g) CNN-DMRI; (h) RicianNet; (i) 2D-DCRNet; (j) 2D-Parallel-RicianNet; (k) 3D-DCRNet; (l) 3D-Parallel-RicianNet.

**Figure 8:**
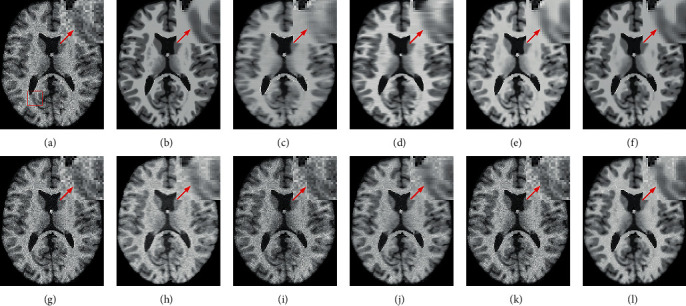
Denoising effect of different methods with 9% noise level. (a) Noisy image; (b) noise-free image; (c) NLM; (d) BM3D; (e) ODCT3D; (f) PRI-NLM3D; (g) CNN-DMRI; (h) RicianNet; (i) 2D-DCRNet; (j) 2D-Parallel-RicianNet; (k) 3D-DCRNet; (l) 3D-Parallel-RicianNet.

Figures 7 and 8 provide a visual comparison for T1w images from testing data under 3% and 9% noise levels using 10 methods. The zoomed-in regions of the denoised images are shown to observe noticeable details. In Figure 7, all methods can achieve good performance under low-level noise circumstances. However, traditional methods suffer from obvious oversmoothing effects and distort some important details. Among deep learning methods, the images processed by CNN-DMRI, 2D-DCRNet, 2D-Parallel-RicianNet, and 3D-DCRNet have obvious Rician noise. RicianNet increases the brightness of the brain area and makes it difficult to clearly observe the anatomical structure. Figure 7 shows that the 3D-Parallel-RicianNet denoising method gives better results and preserves the key information in the image.

While the noise level increases, the traditional methods suffer from obvious oversmoothing effects, as shown in Figure 8. CNN-MRI and RicianNet models still have some noise and suffer from slight oversmoothing of textured regions. By using the DCR module, 2D-DCRNet and 3D-DCRNet have a strong denoising ability globally for 2D slice-based and 3D patch-based cases. However, without considering local structural features, the DCRNet model loses some important local details in the denoising process. Hence, by combining global features of DCRNet and local features of DSCRNet, the proposed 3D-Parallel-RicianNet can preserve finer detailed structures in homogeneous areas, and it obtains the most consistent results with noise-free images. Hence, our 3D-Parallel-RicianNet method can better retain the key information in denoised MR images, which is useful for improving the precision of clinician diagnosis.

### 3.5. Clinical Results

#### 3.5.1. Results from the IXI-Hammersmith Dataset

To validate the performance of the proposed 3D-Parallel-RicianNet, ten denoising methods were compared on different clinical data sets.

**Figure 9:**
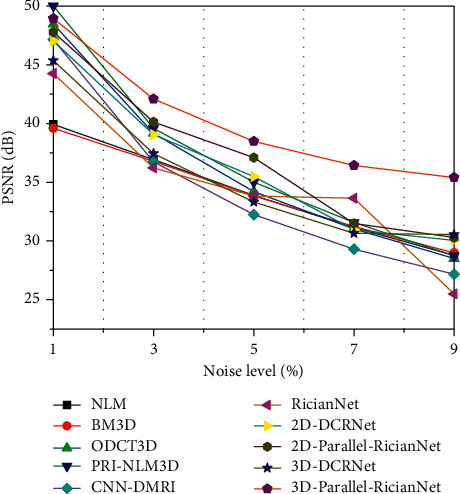
PSNRs using 10 methods under different noise levels for the IXI-Hammersmith dataset.

**Figure 10:**
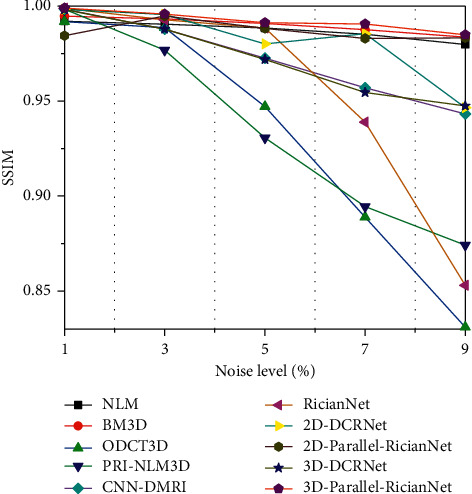
SSIMs using 10 methods under different noise levels for the IXI-Hammersmith dataset.

**Figure 11:**
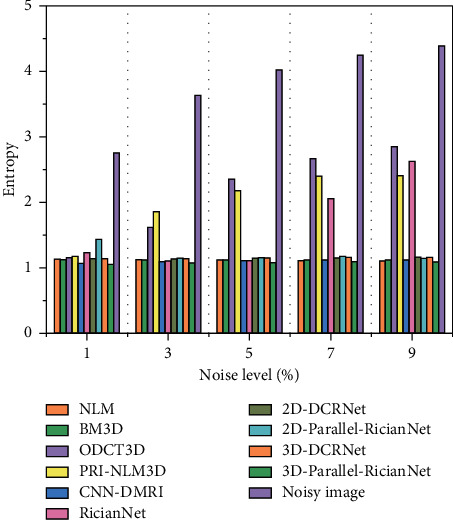
Entropy using 10 methods under different noise levels for the IXI-Hammersmith dataset.

Figures 9–11 summarize the three metrics in the IXI-Hammersmith dataset with 10 methods under different noise levels. At a noise level of 1%, the PRI-NLM algorithm achieves denoising performance comparable to that of 3D-Parallel-RicianNet in terms of PSNR. At noise levels above 5%, the proposed model produces higher PSNRs than the competing methods. In particular, in Figure 10, we can see that the 3D-Parallel-RicianNet model consistently yields SSIMs higher than the other nine methods for all noise levels. From the perspective of entropy, our method had a low entropy value. These results indicated that the 3D-Parallel-RicianNet model had a strong denoising ability.

**Figure 12:**
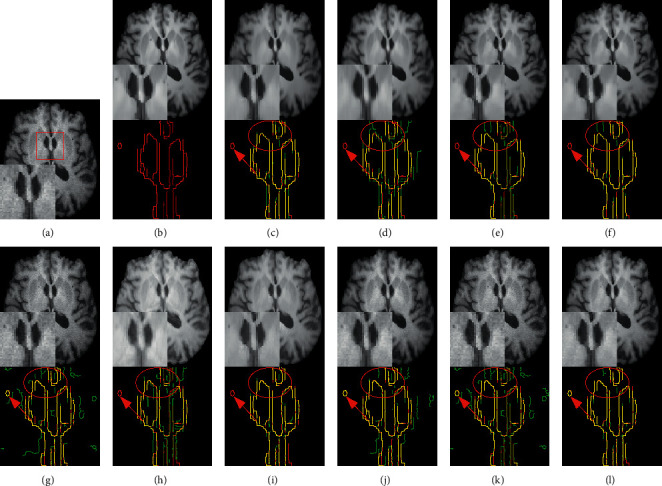
The denoising effect for IXI-Hammersmith dataset with different methods at 3% noise level. (a) Noisy image; (b) noise-free image; (c) NLM; (d) BM3D; (e) ODCT3D; (f) PRI-NLM3D; (g) CNN-DMRI; (h) RicianNet; (i) 2D-DCRNet; (j) 2D-Parallel-RicianNet; (k) 3D-DCRNet; (l) 3D-Parallel-RicianNet. Each method below shows the corresponding edge detection image of the enlarged area (green), and the yellow represents the overlapping area with the noise-free image (red).

Figure 12 shows an example of denoising results using 10 methods on the IXI-Hammersmith dataset with 3% noise. It can be seen in the figure that the proposed 3D-Parallel-RicianNet model gives the best denoising results and the denoised image is virtually identical to the ground-truth image. After visual inspection, it can be deduced that the outcome of our proposed 3D-Parallel-RicianNet is improved compared to the others in terms of fine-structure retention and edges.

#### 3.5.2. Results from the IXI-Guys Dataset

**Figure 13:**
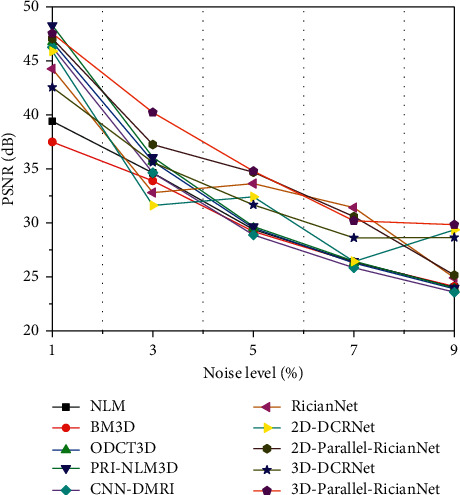
PSNRs using 10 methods under different noise levels for the IXI-Guys dataset.

**Figure 14:**
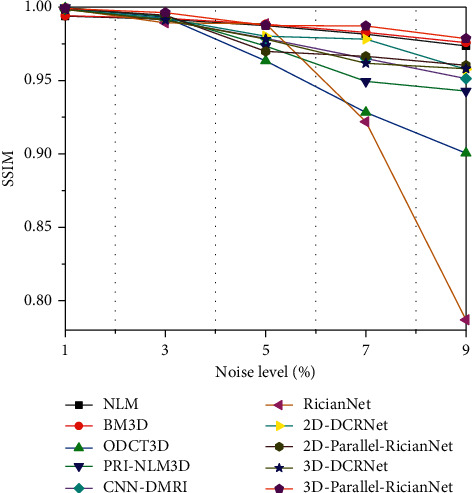
SSIMs using 10 methods under different noise levels for the IXI-Guys dataset.

**Figure 15:**
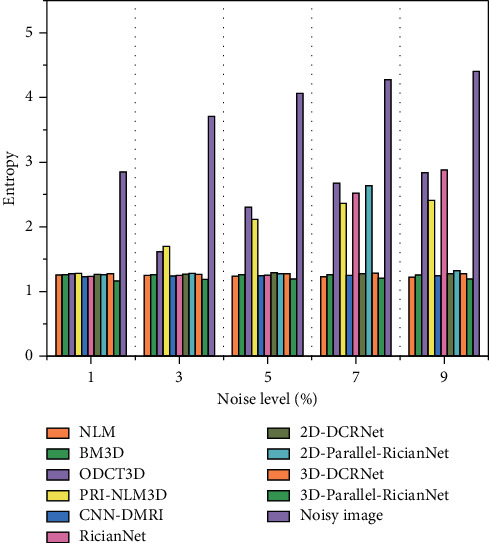
Entropy using 10 methods under different noise levels for the IXI-Guys dataset.

Figures 13–15 summarize the PSNR, SSIM, and entropy values using 10 methods on the IXI-Guys dataset. We test the trained model with the IXI-Hammersmith dataset on this IXI-Guys dataset, which reflects network generalization on other nontrained datasets. The 3D-Parallel-RicianNet shows the most robust performance among the tested methods in terms of PSNR, SSIM, and entropy. In particular, our model still achieves better denoising ability than other methods at higher noise levels.

**Figure 16:**
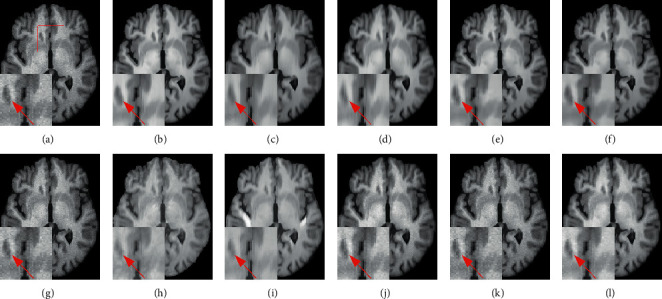
The denoising effect for IXI-Guys dataset with different methods at 3% noise level. (a) Noisy image; (b) noise-free image; (c) NLM; (d) BM3D; (e) ODCT3D; (f) PRI-NLM3D; (g) CNN-DMRI; (h) RicianNet; (i) 2D-DCRNet; (j) 2D-Parallel-RicianNet; (k) 3D-DCRNet; (l) 3D-Parallel-RicianNet.

Figure 16 shows an example of denoising results obtained with 10 methods on data from the IXI-Guys dataset at the 3% noise level. Consistent with the denoising performance on the IXI-Hammersmith dataset, the proposed 3D-Parallel-RicianNet method provided the best denoising result and removed the image noise more robustly than the other methods on the IXI-Guys dataset. Particularly in the region indicated by the red line, the 3D-Parallel-RicianNet model achieved better visual results.

#### 3.5.3. Results from the ADNI Dataset

**Figure 17:**
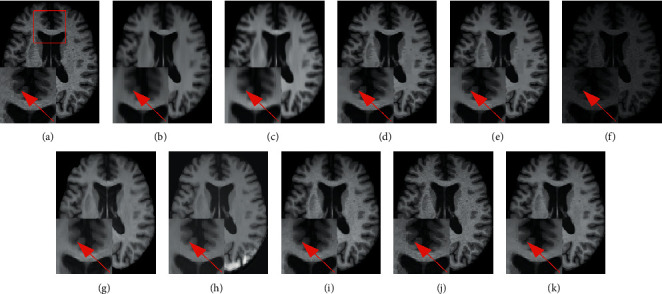
The denoising effect of ADNI set with different methods at 3% noise level. (a) Noisy image; (b) NLM; (c) BM3D; (d) ODCT3D; (e) PRI-NLM3D; (f) CNN-DMRI; (g) RicianNet; (h) 2D-DCRNet; (i) 2D-Parallel-RicianNet; (j) 3D-DCRNet; (k) 3D-Parallel-RicianNet.

**Figure 18:**
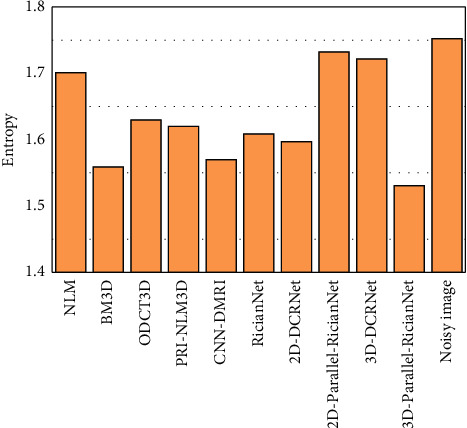
The entropy of the denoised images from the ADNI database.

This subsection is devoted to verifying the consistency of the proposed approach on the ADNI dataset. Because noise-free images are unavailable, entropy is measured and used as the quantitative metric. The results are shown in Figures 17 and 18.

As shown in Figure 17, although the RicianNet and 2D-DCRNet remove noise, they suffer from obvious oversmoothing effects, and it is difficult to identify the key anatomical structures. In addition, the denoising effect is not satisfactory when using BM3D, ODCT3D, PRI-NLM3D, CNN-DMRI, 2D-Parallel-RicianNet, and 3D-DCRNet. The results of these methods still contain substantial noise and miss some of the structural details. It can be noted that 3D-Parallel-RicianNet retains the details better than other methods.

According to Figure 18, the entropy results of denoised MR images in the ADNI dataset using different processing methods are compared. We find that 3D-Parallel-RicianNet achieves the lowest entropy value. Combined with Figure 17, we find that our method not only effectively removes noise but also preserves more useful key information in images. Hence, our 3D-Parallel-RicianNet method has strong generalization ability and strong robustness. These experimental results once again demonstrate the advantages of our proposed model.

#### 3.5.4. Denoising of Real Abdominal MR Data

In this subsection, we performed denoising for abdominal MR images by the proposed network. We compared three denoising methods, and the experimental results are shown in Table 6.

**Figure 19:**
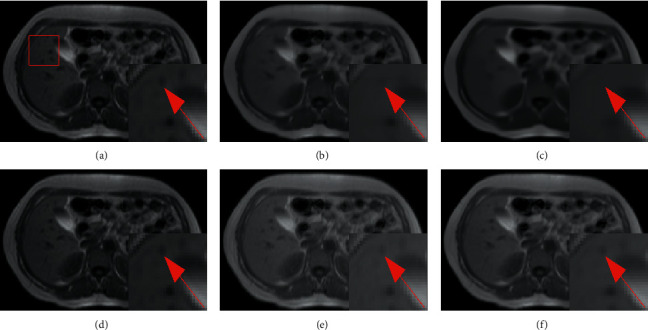
The denoising effect of CHAOS set with different methods at 3% noise level. (a) Noisy image; (b) noise-free image; (c) BM3D; (d) CNN-DMRI; (e) RicianNet; (f) 3D-Parallel-RicianNet.

Table 6 shows that the PSNR of our method can reach 39.7090, which is higher than those of BM3D, CNN-DMRI, and RicianNet. On SSIM, RicianNet is lower than BM3D and CNN-DMRI, indicating that although RicianNet can remove noise, it cannot retain the structure information of the image. Our method can still obtain the highest SSIM value. We show the denoising results of the four methods in Figure 19. It can be seen from the figure that our method can not only remove noise but also preserve the key anatomical position information in the image completely.

### 3.6. Comparisons of the Results with Different Spatial Resolutions

The image resolution affects the quality of the image. Generally, when the image resolution is smaller, the denoising ability of the model is significantly reduced. In this part, we use the BrainWeb dataset to verify the denoising effect of images with different resolutions at a noise level of 3%. The results are shown in Table 7.

From Table 7, it can be observed that the proposed 3D-Parallel-RicianNet outperforms other methods tested among the different spatial resolutions. For BM3D and RicianNet, they are difficult to remove noise at low spatial resolutions. It is noted that noise cleaning appears to have a consistent effect when different spatial resolutions are relatively close, such as 0.9375 × 0.9375 × 0.9375mm^3^ and 1 × 1 × 1mm^3^. However, it should be noted that some loss of contrast and spatial resolution is possible. Once the difference between the resolutions becomes larger, the denoising effect will also change significantly, such as 1 × 1 × 1mm^3^ and 2 × 2 × 2mm^3^. In addition, the PSNRs of deep learning methods decrease significantly with decreasing spatial resolution, while the SSIM values are relatively close, indicating that deep learning methods recover most of the complex anatomical structures. Compared to other methods, our model has a more balanced denoising ability at different spatial resolutions, and the mean value of PSNR can reach 41.6373. Since the proposed 3D-Parallel-RicianNet can extract the global and local features in the noisy image and restore the clean image, it can still maintain denoising ability at low spatial resolution.

### 3.7. Comparisons of the Results with Different Brain Tissues

**Figure 20:**
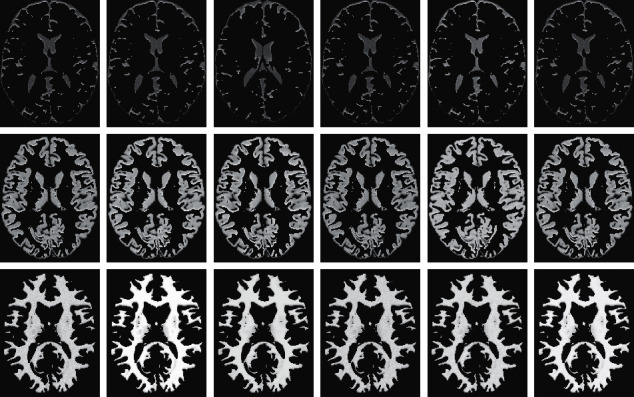
The denoising effect of brain tissues with different methods at 3% noise level. Noisy image (1^st^ column); noise-free image (2^nd^ column); BM3D (3^rd^ column); CNN-DMRI (4^th^ column); RicianNet (5^th^ column); 3D-Parallel-RicianNet (6^th^ column).

Based on MR imaging technique, key brain tissues like gray matter (GM), white matter (WM), and cerebrospinal fluid (CSF) become visible. These three tissues help visualize brain structures and guide surgery but noise can affect the interpretation of brain tissue [58]. To evaluate the denoising effectiveness of 3D-Parallel-RicianNet on different brain tissues, state-of-the-art methods BM3D, CNN-DMRI, and RicianNet are compared in Table 8. The proposed model can achieve better PSNR and SSIM results than the competing methods in different brain tissues. In particular, in CSF, we can see that the PSNR of 3D-Parallel-RicianNet can reach 51.9105. We also show the different brain tissue denoising results of four denoising methods in Figure 20. These experimental results once again demonstrate the advantages of the proposed model.

### 3.8. Variants of the **R** Setting in the DCR Module

In our model, the DCR module of different *R* is our key component. The PSNR and SSIM are recorded in Table 9 by different *R* settings at the 3% noise level in the BrainWeb dataset. We conducted three experiments, each using the same dilation rate for the 18 DCR modules. The final receptive fields are 37, 73, and 109. Combining Tables 1 and 2 and Table 8, we can find that our hybrid dilation rate can reach the highest PSNR and SSIM. When *R*=2 and 3, the receptive field has already caused waste. In addition, using the same dilation rates can easily cause gridding effects. There is a lack of correlation between the feature maps extracted in this way, and an accurately predicted result cannot be obtained in the end. Therefore, our model can achieve superior denoising performance.

## 4. Discussion and Conclusions

In this work, we propose a parallel denoising residual network based on cascaded DCR and DSCR modules to address the random noise in MR images. The global and local features are extracted by the designed DCRNet and DSCRNet, and then these features are fused together. Hence, global and local information is captured to drive the denoising progress of brain MR images by supervised network learning.

The PSNR, SSIM, and entropy are calculated to compare the proposed method with many existing methods, and the denoising effect of the proposed method is verified on the BrainWeb simulation data under different noise levels. To test the practicability of the proposed network, experiments on real clinical MR images show that the proposed method is superior to other methods for the IXI-Hammersmith, IXI-Guys, ADNI, and CHAOS datasets.

In this work, one of our limitations is that although structural information can be retained at high noise levels, there is still a small amount of local noise, as shown in Figure 8. Next, we will continue to study to find a balance between noise removal and structure maintenance at different noise levels. Another critical limitation of our method is the requirement for high-quality noise-free ground-truth images, which are difficult to obtain in real applications. Incorporation of prior knowledge about organ shape and location is key to improving the performance of image analysis approaches. However, in most recently developed medical image analysis techniques, it is not obvious how to incorporate such prior knowledge [59]. Oktay et al. incorporated anatomical prior knowledge into a deep learning method through a new regularization model, and this method showed that the approach can be easily adapted to different medical image analysis tasks (e.g., image enhancement and segmentation) [59]. Furthermore, in [60], the author used morphological component analysis (MCA) to decompose noisy images into cartoon, texture, and residual parts that were considered noise components. Therefore, to circumvent the limitations of our method, we will verify it using multimodality images and incorporate other meaningful priors, such as residual parts, organ shape, and location to mitigate semisupervised denoising tasks in the future.

In conclusion, the results obtained in this paper are encouraging and efficiently demonstrate the potential of our 3D-Parallel-RicianNet method for MR image denoising. This method can not only effectively remove noise in MR images but also preserve enough detailed structural information, which can help to provide high-quality MR images for clinical diagnosis.

## Figures and Tables

**Table 1:** PSNRs on different noise levels from BrainWeb dataset with 10 methods.

Methods	1%	3%	5%	7%	9%
NLM	34.1381	32.1259	29.9717	29.4599	27.8319
BM3D	35.3151	33.1937	31.0144	30.4890	28.9093
ODCT3D	48.1295	36.2756	31.5857	30.5124	28.2452
PRI-NLM3D	49.8923	36.8192	31.9597	30.9418	28.5961
CNN-DMRI	47.8125	35.4720	30.8958	29.3533	27.2152
RicianNet	43.4145	37.0095	27.4807	27.6777	28.8616
2D-DCRNet	46.8168	38.6786	34.6277	32.4664	30.5232
2D-Parallel-RicianNet	50.9072	41.8099	38.8218	35.9767	34.5207
3D-DCRNet	48.5120	39.1336	35.1466	32.7280	31.0916
3D-Parallel-RicianNet	**51.7192**	**43.9950**	**40.9218**	**37.6896**	**37.1069**

**Table 2:** SSIMs on different noise levels from BrainWeb dataset with 10 methods.

Methods	1%	3%	5%	7%	9%
NLM	0.9669	0.9605	0.9539	0.9521	0.9454
BM3D	0.9768	0.9725	0.9673	0.9649	0.9584
ODCT3D	0.9992	0.9959	0.9903	0.9857	0.9778
PRI-NLM3D	0.9995	0.9967	0.9922	**0.9889**	0.9823
CNN-DMRI	0.9987	0.9891	0.9727	0.9533	0.9312
RicianNet	0.9606	0.8906	0.9221	0.7246	0.6319
2D-DCRNet	0.9986	0.9900	0.9906	0.9535	0.9346
2D-Parallel-RicianNet	0.9992	0.9933	0.9864	0.9685	0.9722
3D-DCRNet	0.9985	0.9898	0.9587	0.9561	0.9373
3D-Parallel-RicianNet	**0.9995**	**0.9982**	**0.9942**	0.9883	**0.9859**

**Table 3:** Entropy on different noise levels from BrainWeb dataset with 10 methods.

Methods	1%	3%	5%	7%	9%
Noisy image	2.4787	2.5067	2.5266	2.5482	2.5556
NLM	2.4721	2.4581	2.4474	2.4470	2.4324
BM3D	2.4532	2.4476	2.4849	2.4676	2.4803
ODCT3D	2.4516	2.4844	2.4988	2.5102	2.5033
PRI-NLM3D	2.4447	2.4415	2.4291	2.4330	2.4201
CNN-DMRI	2.4499	2.4869	2.5086	2.5313	2.5406
RicianNet	2.4629	2.4715	2.4519	2.4511	2.4606
2D-DCRNet	2.4624	2.4701	2.4742	2.3481	2.4142
2D-Parallel-RicianNet	2.4693	2.3995	2.4073	2.4631	2.1374
3D-DCRNet	2.4556	2.4488	2.4331	2.3340	2.1787
3D-Parallel-RicianNet	**2.4364**	**2.3469**	**2.3275**	**2.2890**	**2.0642**

**Table 4:** Execution time on different noise levels from BrainWeb dataset with 10 methods.

Methods	1%	3%	5%	7%	9%	Average
NLM	10.55	10.54	10.58	10.54	10.55	10.55
BM3D	28.26	28.13	27.29	27.37	28.25	27.86
ODCT3D	23.54	17.56	18.02	14.75	14.54	17.68
PRI-NLM3D	14.07	12.50	12.92	13.59	13.28	13.27
CNN-DMRI	1.13	1.11	1.13	1.12	1.12	1.12
RicianNet	1.71	1.65	1.65	1.69	1.66	1.67
2D-DCRNet	1.27	1.32	1.34	1.33	1.29	1.31
2D-Parallel-RicianNet	1.18	1.14	1.13	1.12	1.12	1.14
3D-DCRNet	0.94	0.92	0.91	0.91	0.92	0.92
3D-Parallel-RicianNet	**0.89**	**0.89**	**0.89**	**0.89**	**0.89**	**0.89**

**Table 5:** Number of parameters of different networks.

Method	CNN-DMRI	RicianNet	3D-Parallel-RicianNet
Number of parameters	1,444,929	5,346,114	395,405

**Table 6:** The PSNR and SSIM on different noise levels from CHAOS dataset with 4 methods.

Method	BM3D	CNN-DMRI	RicianNet	3D-Parallel-RicianNet
PSNR	31.9167	32.0655	35.2577	**39.7090**
SSIM	0.9862	0.9867	0.9258	**0.9941**

**Table 7:** PSNR (top) and SSIM (bottom) comparisons of different algorithms with different spatial resolutions.

Spatial resolutions (mm^3^)	Method			
	BM3D	CNN-DMRI	RicianNet	3D-Parallel-RicianNet
0.9 × 0.9 × 0.9	31.8908 0.9709	34.7330 0.9890	35.7466 0.9233	41.5169 0.9970
0.9375 × 0.9375 × 0.9375	32.1794 0.9718	35.2350 0.9903	36.4030 0.9117	41.9756 0.9973
1 × 1 × 1	33.1937 0.9725	35.4720 0.9891	37.0095 0.8906	43.9950 0.9982
1.1 × 1.1 × 1.1	31.7986 0.9677	35.6006 0.9917	36.6172 0.9078	42.2193 0.9976
1.25 × 1.25 × 1.25	31.3591 0.9633	34.0402 0.9917	36.0542 0.9070	41.9259 0.9974
1.5 × 1.5 × 1.5	29.8656 0.9481	35.1568 0.9918	31.4012 0.9180	40.3297 0.9961
2 × 2 × 2	28.2362 0.9230	34.5695 0.9900	23.4731 0.9038	38.4990 0.9933

**Table 8:** PSNR and SSIM comparisons of different methods in different brain tissues.

Method	CSF		GM		WM	
	PSNR	SSIM	PSNR	SSIM	PSNR	SSIM
BM3D	27.0765	0.9213	19.1709	0.9114	18.2741	0.9350
CNN-DMRI	45.8322	0.9981	39.1790	0.9976	38.7420	0.9973
RicianNet	44.8454	0.9982	41.9853	0.9988	43.6082	0.9986
3D-Parallel-RicianNet	**51.9105**	**0.9996**	**47.5288**	**0.9996**	**48.6238**	**0.9998**

**Table 9:** PSNR and SSIM comparisons in different *R* settings.

	*R*=1	*R*=2	*R*=3
PSNR	41.0253	40.4788	39.3627
SSIM	0.9956	0.9661	0.9946

## Data Availability

The BrainWeb, IXI, ADNI, and CHAOS datasets are publicly available (BrainWeb, https://brainweb.bic.mni.mcgill.ca/brainweb/; IXI, http://brain-development.org/ixi-dataset/; ADNI, http://adni.loni.usc.edu/; and CHAOS, https://chaos.grand-challenge.org/).
